# Evaluation of a Non-Stagnant Water Gap in Hollow-Fiber Membrane Distillation and Multistage Performance Limitations

**DOI:** 10.3390/membranes15090253

**Published:** 2025-08-27

**Authors:** Mohamed O. Elbessomy, Kareem W. Farghaly, Osama A. Elsamni, Samy M. Elsherbiny, Ahmed Rezk, Mahmoud B. Elsheniti

**Affiliations:** 1 Mechanical Engineering Department, Faculty of Engineering, Alexandria University, El-Chatby, Alexandria 21544, Egypt; mohamed.elbessomy@alexu.edu.eg (M.O.E.); kareem-wagdy@alexu.edu.eg (K.W.F.); elsamni@alexu.edu.eg (O.A.E.); samy.elsherbiny@alexu.edu.eg (S.M.E.); 2 Mechanical, Biomedical and Design Engineering Department (MBDE), College of Engineering and Physical Science, Aston University, Birmingham B4 7ET, UK; 3 Energy and Bioproducts Research Institute (EBRI), College of Engineering and Physical Science, Aston University, Birmingham B4 7ET, UK; 4 Mechanical Engineering Department, College of Engineering, King Saud University, Riyadh 11451, Saudi Arabia

**Keywords:** membrane distillation, distillate water flux, numerical simulation, water gap circulation, multistage configuration

## Abstract

Hollow-fiber water gap membrane distillation (HF-WGMD) modules are gaining attention for desalination applications due to their compact design and high surface-area-to-volume ratio. This study presents a comprehensive CFD model to analyze and compare the performance of two HF-WGMD module configurations: one with a conventional stagnant water gap (WG) and the other incorporating water gap flow circulation. The model was validated against experimental data, showing excellent agreement, and was then used to simulate flow patterns in the feed, water gap, and coolant domains. Results indicate that, at a feed temperature of 80 °C with a stagnant WG, employing a turbulent flow scheme in the feed side increases water flux by 20.7% compared to laminar flow, while increasing coolant flow rate has a minor impact. In contrast, introducing circulation within the water gap significantly enhances performance, boosting water flux by 30.1%. This effect becomes more pronounced with rising feed temperature: increasing from 50 °C to 80 °C leads to a flux increase from 6.74 to 27.89 kg/(m^2^h) under circulating WG conditions. However, in multistage systems, the energy efficiency trade-off becomes evident. Water gap circulation is more energy-efficient than the stagnant configuration only for systems with fewer than 20 stages. At higher stage counts, the stagnant WG setup proves more efficient. For example, at 80 °C and 50 stages, the stagnant configuration consumes just 793 kWh/m^3^, representing a 47.3% reduction in energy consumption compared to the circulating WG setup. These findings highlight the performance benefits and energy trade-offs of water gap circulation in HF-WGMD systems, providing valuable guidance for optimization and scalability of high-efficiency desalination module designs.

## 1. Introduction

Membrane distillation is a thermally driven separation process that leverages vapor pressure differences to facilitate the diffusion of water vapor through a hydrophobic membrane, effectively isolating salts and other impurities. The technique is adaptable to various configurations, each suited to specific applications and offering distinct advantages, such as direct contact membrane distillation (DCMD) [1,2], air gap membrane distillation (AGMD) [3,4], sweeping gas membrane distillation (SGMD) [5,6], and vacuum membrane distillation (VMD) [7,8].

Water gap membrane distillation (WGMD) has emerged as a promising technology for water desalination. In a conventional WGMD configuration, a stagnant layer of distillate water is maintained on the cold side of a hydrophobic membrane, serving as the permeate for the module. Additionally, a cooling fluid is separated from the permeate by a cooling plate, ensuring that the water gap receives adequate cooling. This configuration enhances the water flux of WGMD compared to air gap membrane distillation (AGMD) systems [9,10,11,12]. Furthermore, WGMD modules exhibit superior thermal efficiency, resulting in lower thermal energy consumption when compared to direct contact membrane distillation (DCMD) modules [13,14].

Numerous studies have investigated the conventional WGMD configuration as a viable technology for desalinating saline water, focusing on enhancing the performance of various WGMD modules [15,16,17]. Lawal et al. [18] conducted experimental investigations to assess the impact of different cooling plate materials on the performance of plate and frame WGMD modules under various feed and coolant operating conditions. In their experiments, the water gap thickness was maintained at 5 mm, the module length at 40 mm, and seawater salinity at the feed inlet was considered for all test cases. The results indicated that increasing the thermal conductivity of the cooling plate positively influenced the module’s water flux, achieving a maximum water flux of 32 kg/(m^2^h) when utilizing a copper cooling plate (the most conductive material studied) at feed and coolant inlet temperatures of 70 °C and 15 °C, respectively, with a feed flow rate of 1.2 L/min. The gained output ratio (GOR) of the module was approximately 0.38. Furthermore, reducing the feed flow rate improved the thermal performance of the module, resulting in a GOR of 0.42 at a flow rate of 0.6 L/min, under the same temperature conditions. Notably, the use of a stainless steel plate yielded a slightly higher GOR of about 0.43 under identical operating conditions. Elsheniti et al. [13] conducted a comparative numerical study between DCMD and WGMD hollow-fiber (HF) modules. They developed a two-dimensional axisymmetric transient computational fluid dynamics (CFD) model to simulate the concentrating process of feed water while recirculating through the modules’ feed channels. The study was performed at three different feed tank temperature levels, maintaining the effective module length at 100 mm, with an initial feed salinity of 70,000 ppm and permeate (for DCMD) and coolant (for WGMD) inlet temperatures set at 20 °C. The findings indicated that the WGMD desalination system achieved an average water flux of 8.85 kg/(m^2^h). In comparison, the DCMD system demonstrated a 25.4% increase in water flux at a feed tank temperature of 70 °C while concentrating feed water to 233,333 ppm. However, WGMD demonstrated superior energy efficiency, with specific thermal energy consumption (STEC) recorded at 903 kWh/m^3^, compared to 1026 kWh/m^3^ for the DCMD system under similar conditions while concentrating feed water to 100,000 ppm. The GOR for WGMD was 0.72, whereas it was 0.63 for DCMD at the same operational parameters.

Several studies have explored unconventional WGMD modules, such as material gap or conductive gap membrane distillation modules [19,20,21], aimed at enhancing the transport properties of the module gap. Other research efforts have proposed the incorporation of external sources to improve the characteristics of the conventional water gap, such as the use of a rotating impeller [22,23]. For instance, Lawal [22] experimentally examined the performance of a WGMD module equipped with a circulation impeller placed within the water gap to enhance transport characteristics. The study evaluated the effect of impeller rotation speed on the performance of a module with a length of 90.25 mm and a gap thickness of 11 mm at a salinity of 4080 ppm. The results indicated that increasing the impeller speed up to 1100 rev/min significantly improved the overall performance of the module, with a 153.1% increase in water flux compared to the conventional WGMD module with a stagnant water gap at feed and coolant inlet temperatures of 70 °C and 20 °C, respectively. Moreover, the STEC of the module was reduced to 1400 kWh/m^3^, achieving a 12.2% reduction compared to the conventional WGMD module under the same temperature conditions.

Despite ongoing research aimed at enhancing the thermal performance of WGMD systems, significant improvements are still needed. One effective strategy for reducing thermal energy consumption in WGMD modules involves implementing a multistage (MS) arrangement, which incorporates multiple modules in series [24,25,26]. A series connection enables the feed water to be expelled at lower temperature levels while maximizing the distillate water extraction. In the meanwhile, thermal energy gained through the coolant channel is recovered to preheat saline water before it enters the feed channel. For instance, Alawad et al. [27] experimentally investigated the influence of various operating conditions, including feed and coolant inlet temperatures and flow rates, on the thermal performance of a multistage WGMD desalination system. This MS-WGMD system, consisting of four stages arranged in series, was evaluated with a fixed feed inlet salinity of 250 ppm. The study revealed that the four-stage system achieved an STEC of 1543 kWh/m^3^, representing a 50.6% reduction in STEC compared to a single module at feed and coolant inlet temperatures of 70 °C and 25 °C, respectively. Additionally, the GOR was measured at 0.43, indicating an increase of 104.8% over that of the single module under the same feed and coolant inlet temperatures. As another tactic to make the module more compact, Elbessomy et al. [28] examined the impact of helical configurations of single and double hollow fibers inserted within the cooling tubes on the productivity and thermal performance. The results indicated that single helical fiber modules enhanced water flux by approximately 11.4% at feed and coolant inlet temperatures of 70 °C and 20 °C, respectively, while double helical fibers showed only an 8.07% increase under the same conditions. Furthermore, the study demonstrated that increasing fiber length through helical configuration could reduce the module’s STEC from 6000 kWh/m^3^ for a straight single fiber to 3900 kWh/m^3^ for a single fiber with 50 helical turns. Notably, employing up to three stages of helical single fiber modules in series could further decrease the desalination system’s STEC to 1800 kWh/m^3^.

A review of the literature reveals a notable gap in exploring the potential superiority of a circulating water gap over a stagnant process, especially concerning the scaling up of hollow-fiber membrane distillation. Developing and validating a CFD simulation model can offer several important benefits. It allows for flexible and detailed visualization of system performance across complex geometries and a wide range of design and operating conditions. Furthermore, it significantly reduces the time and cost typically associated with experimental setup and testing.

To date, most studies focus on conventional stagnant water gap configurations, overlooking the impact of circulation in the water gap on the hollow-fiber membrane performance. To address this gap, this study develops a theoretical framework and a CFD simulation to implement and analyze the circulating water gap in HF-WGMD modules to be compared with conventional modules. A two-dimensional axisymmetric mathematical model is established, incorporating mass, momentum, and energy conservation equations to simulate the transport phenomena across the feed channel, membrane, water gap, cooling tube, and coolant stream. The model provides a detailed assessment of temperature and concentration distributions, offering new insights into the influence of water gap circulation against conventional stagnant water gap configuration. A parametric study evaluates key operational factors, including feed and coolant velocities, temperatures, and the circulating water gap flow rate. Unlike previous studies [13,15,28], the current work investigates the influence of turbulent flow regime for the different module streams on the HF-WGMD module productivity. Furthermore, the performance of a multistage circulating HF-WGMD module is evaluated and compared with that of a conventional stagnant configuration. Comparative analyses based on water flux and STEC are carried out to explore the limitations of water gap circulation in enhancing module productivity and thermal efficiency, offering new insights into the design and optimization of high-performance HF-WGMD systems.

## 2. Stagnant and Circulating Multistage HF-WGMD Systems’ Layout

**Figure 1:**
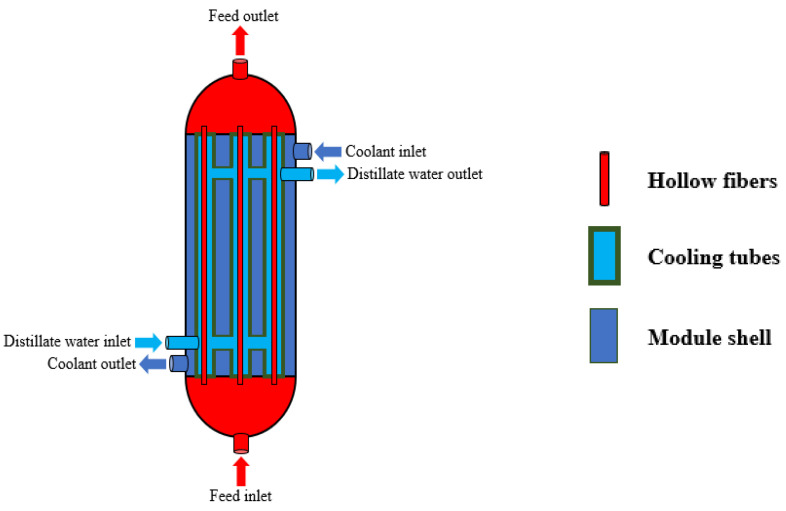
Schematic diagram of the HF-WGMD module with a circulating water gap.

The HF-WGMD desalination module adopted in the current study features a shell-and-tube configuration, where the feed channels are created by the lumen sides of the hollow fibers within the module. Each hollow fiber is positioned centrally within a cooling tube, which establishes the module distillate water gap. Concurrently, cooling water flows through the shell side of the module. A schematic diagram of the HF-WGMD module is illustrated in Figure 1.

**Figure 2:**
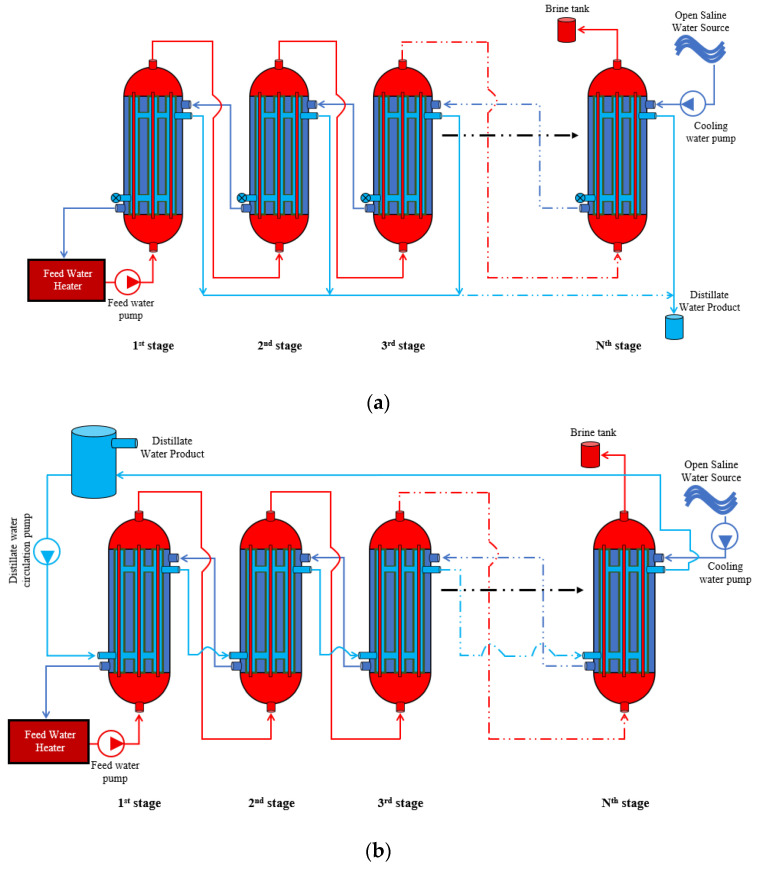
Schematic diagrams for the configurations of multistage HF-WGMD modules with (**a**) a stagnant WG and (**b**) a circulating WG.

Two configurations of the MS-HF-WGMD module are investigated: the stagnant water gap and circulating water gap. The stagnant module is illustrated in Figure 2a, where several HF-WGMD modules are configured in series, with stagnant water in modules’ WG. Cooling water, drawn from an open saline water source, is directed through the shell side of the modules, flowing from the last module to the first. This arrangement enables the recovery of thermal energy from the feed channels via the saline water before it enters the primary feed water heater. Subsequently, the feed water traverses the lumen side of the hollow fibers in the modules, starting from the first module and exiting into a rejected brine tank from the last module, thereby achieving a counter-current flow pattern for both feed and coolant. The distillate water product is collected via overflow from the stagnant water gaps.

On the other hand, Figure 2b depicts a similar feed and coolant flow arrangement for the MS-HF-WGMD module, but with circulation in the water gap. In this configuration, the distillate water is circulating through the gap between the cooling tubes and the hollow fibers of the modules, following a concurrent flow pattern with the feed water. The distillate water product is then collected in a common water tank. Under steady-state condition, the temperature at the inlet of the circulating water gap is equal to that at the outlet of the water gap in the final stage, since the circulating water is recirculated in a closed loop, while the product is continuously withdrawn from the distillate tank, as illustrated in Figure 2b. In the model, this was implemented by applying a periodic boundary condition to these two boundaries.

## 3. Methodology

A 2D axisymmetric steady-state model is introduced in this study to simulate the mass, momentum, and heat transport physics in HF-WGMD systems with both stagnant and circulation water gap configurations. The model is constructed using the fundamental principles of mass, momentum, and energy conservations.

To simplify the computational process while preserving solution accuracy, the considered assumptions are as follows:

- The system remains at a steady-state condition throughout all investigations.
- The hollow-fiber membrane and cooling tube are perfectly concentric.
- The hollow-fiber membrane exhibits uniform and isotropic porosity.
- Fouling at the feed–membrane interface is neglected.
- Pore wetting within the membrane is assumed to be absent.
- Heat loss from the WGMD module is considered negligible.

**Figure 3:**
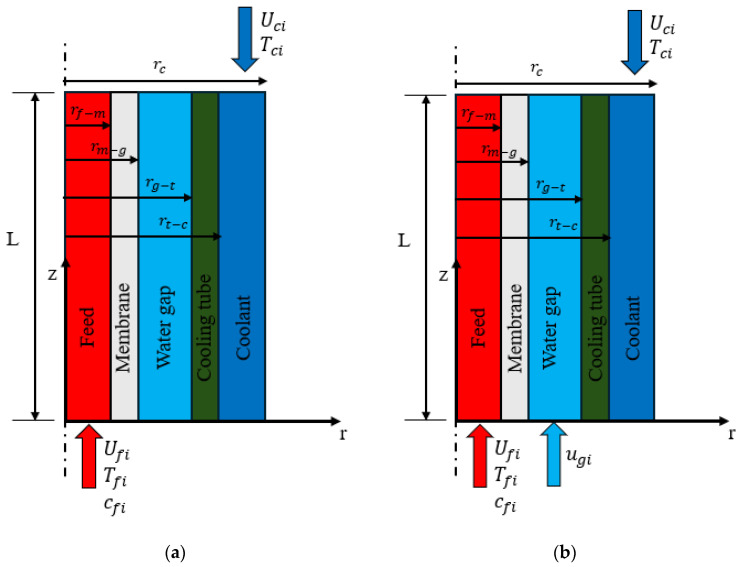
Schematic diagrams of the simulated domains of HF-WGMD modules; (**a**) a stagnant WG and (**b**) a circulating WG.

The model includes five domains entitled the feed channel, hollow-fiber membrane layer, water gap, cooling tube, and cooling channel, as depicted in Figure 3. However, the distillate water is considered stagnant in the water gap, as shown in Figure 3a, it is circulated using an external water pump as shown in Figure 3b. It should also be noted that, in the multistage configuration, all modules are connected in series. Consequently, the feed, stagnant or circulating water gap, and coolant streams can be equivalently represented by a single extended HF-WGMD module, whose effective length equals the length of one module multiplied by the number of stages. Table 1 outlines the defined dimensional parameters and operational conditions of the primary module, offering a detailed summary of the system’s specifications.

### 3.1. Governing Equations

#### 3.1.1. Mass Transport

The concentration balance is applied for feed and membrane domains to determine the distribution of water concentration. In the feed domain, both convection and diffusion terms are considered in Fick’s law, which is mathematically described as follows:

(1) $$u_{f}\frac{\partial c_{f}}{\partial r}+w_{f}\frac{\partial c_{f}}{\partial z}=\frac{1}{r}\frac{\partial }{\partial r}\left(D_{w}r\frac{\partial c_{f}}{\partial r}\right)+\frac{\partial }{\partial z}\left(D_{w}\frac{\partial c_{f}}{\partial z}\right)$$

where $c_{f}$ represents the water concentration within the feed channel, $u_{f}$ and $w_{f}$ denote the velocity components of the feed flow in the radial (r) and axial (z) directions, respectively. The mutual diffusion coefficient of water and salt, $D_{w}$, is determined using the Wilke–Chang equation [29]:

(2) $$D_{w}=\frac{7.4\times 10^{-8}{\left(2.6M_{f}\right)}^{1/2}{(T}_{f}+273)}{\mu_{f}\;{V_{w}}^{0.6}}$$

where $T_{f}$ is the feed water local temperature, $M_{f}$ represents the molecular mass of feed solution, $\mu_{f}$ is the dynamic viscosity of the feed fluid in cP, and $V_{w}$ is the molecular volume of water in cm^3^/mol.

Conversely, within the membrane domain, mass transport is governed exclusively by diffusion only, as water vapor molecules pass through the membrane pores via the diffusion mechanism. This is modeled using Fick’s law as follows:

(3) $$\frac{1}{r}\frac{\partial }{\partial r}\left(D_{m}r\frac{\partial c_{m}}{\partial r}\right)+\frac{\partial }{\partial z}\left(D_{m}\frac{\partial c_{m}}{\partial z}\right)=0$$

Here, $c_{m}$ is the local vapor concentration in the membrane layer, and $D_{m}$ denotes the diffusion coefficient of water vapor through the membrane.

Diffusion mechanism in hydrophobic membranes is influenced by the characteristics of the transported species in addition to membrane’s intrinsic properties. In MD, the primary transport mechanisms are typically Knudsen diffusion, molecular diffusion, and Poiseuille flow. The effective diffusion mechanisms in the membrane model are identified by calculating the Knudsen number $\left(K_{n}\right)$, a dimensionless parameter that quantifies the dominant transport mechanisms, as expressed by the following equation [30]:

(4) $$Kn=\frac{\lambda_{a-w}}{d_{p}}$$

Here, $d_{p}$ is the membrane’s pore diameter, which is equal to 0.16 μm in the current study, while $\lambda_{a-w}$ denotes the mean free path of vapor molecules and can be calculated using the following equation:

(5) $$\lambda_{a-w}=\frac{K_{B}{(T}_{m,avg}+273)}{P\pi {\left[\left(\sigma_{a}+\sigma_{w}\right)/2\right]}^{2}}\times \frac{1}{{\left(1+\left(M_{w}/M_{a}\right)\right)}^{1/2}}$$

In this equation, $T_{m,avg}$ represents the average membrane temperature, P is the pressure, and $K_{B}$ is the Boltzmann constant. The molecular collision diameters for air and water are denoted as $\sigma_{a}=3.71\;A˚$ and $\;\sigma_{w}=2.64\;A˚$, respectively [31]. Additionally, $M_{a}$ and $M_{w}$ represent the molecular masses of air and water, respectively ${(M}_{a}=29\;g/mol$ and $M_{w}=18\;g/mol)$.

In this study, the calculated Knudsen number falls within the transition region $\left(0.1<K_{n}<10\right)$, indicating that the diffusion process is influenced by both Knudsen diffusion and ordinary molecular diffusion. The effective diffusion coefficient is then calculated as the combined contribution of these two mechanisms as follows.

(6) $$D_{or-Kn}={\left(\frac{1}{D_{or}}+\frac{1}{D_{Kn}}\right)}^{-1}$$

(7) $$D_{m}=\frac{\varepsilon }{\tau }D_{or-Kn}$$

where ε indicates the membrane porosity, and τ represents the tortuosity of the membrane pores, which can be estimated using the following equation [32]:

(8) $$\tau =\frac{{\left(2-\varepsilon \right)}^{2}}{\varepsilon }$$

The coefficients for Knudsen diffusion and the ordinary diffusion of vapor in air can be determined using the following equations [30]:

(9) $$D_{or}=1.97\times 10^{-5}{\left(\frac{{(T}_{m}+273)}{256}\right)}^{1.685}$$

(10) $$D_{Kn}=\frac{d_{p}}{3}\times {\left(\frac{8\bar{R}{(T}_{m}+273)}{\pi M_{w}}\right)}^{\frac{1}{2}}$$

#### 3.1.2. Momentum Transport

To obtain the velocity and pressure distributions within the feed, circulating water gap, and cooling channel domains, the governing continuity and Navier–Stokes equations for fluid flow are solved. For a 2D axisymmetric, steady, and incompressible model, these equations are expressed as follows [33]:

(11) $$\frac{1}{r}\frac{\partial (ru)}{\partial r}+\frac{\partial w}{\partial z}=0$$

(12) $$\rho \left[u\frac{\partial u}{\partial r}+w\frac{\partial u}{\partial z}\right]=-\frac{\partial P}{\partial r}+(\mu +\mu_{T})\left[\frac{1}{r}\frac{\partial }{\partial r}\left(r\frac{\partial u}{\partial r}\right)-\frac{u}{r^{2}}+\frac{\partial^{2}u}{\partial z^{2}}\right]$$

(13) $$\rho \left[u\frac{\partial u}{\partial r}+w\frac{\partial w}{\partial z}\right]=-\frac{\partial P}{\partial z}+(\mu +\mu_{T})\left[\frac{1}{r}\frac{\partial }{\partial r}\left(r\frac{\partial w}{\partial r}\right)+\frac{\partial^{2}w}{\partial z^{2}}\right]$$

where ρ and μ are the fluid density and dynamic viscosity, respectively. $\mu_{T}$ is the turbulent viscosity and is set to zero in the laminar flow cases while the realizable k−ε turbulence model, implemented within the COMSOL Multiphysics 5.6 software, is employed to solve for $\mu_{T}$ in the Reynolds-averaged Navier–Stokes (RANS) equations used for turbulent flow cases.

#### 3.1.3. Energy Transport

The temperature distribution is an important factor in the MD process, as it directly impacts the calculations of diffusion coefficients and saturation concentrations. Consequently, the energy conservation equation is solved concurrently with the mass and momentum transport equations to determine the local temperature distribution throughout the entire module. Thermal energy transfer equation, encompassing both convection and conduction, is considered for the feed, circulating water gap, and cooling channels and is expressed as follows:

(14) $$\rho C_{P}\left[u\frac{\partial T}{\partial r}+w\frac{\partial T}{\partial z}\right]=\frac{1}{r}\frac{\partial }{\partial r}\left(kr\frac{\partial T}{\partial r}\right)+\frac{\partial }{\partial z}\left(k\frac{\partial T}{\partial z}\right)$$

where T is temperature, k is the thermal conductivity, and $C_{P}$ is the specific heat of the fluid.

In contrast, only conduction-driven heat transfer is considered in the energy conservation equation for the HF membrane, stagnant water gap, and cooling tube domains and can be presented as follows:

(15) $$\frac{1}{r}\frac{\partial }{\partial r}\left(kr\frac{\partial T}{\partial r}\right)+\frac{\partial }{\partial z}\left(k\frac{\partial T}{\partial z}\right)=0$$

For the membrane layer, the thermal conductivity $\left(k_{m}\right)$ is determined using the following equation:

(16) $$k_{m}=\varepsilon k_{v}+\left(1-\varepsilon \right)k_{s}$$

Here, $k_{s}$ represents the thermal conductivity of the Polyvinylidene Fluoride (PVDF) membrane’s solid matrix. $k_{v}$ denotes the thermal conductivity of water vapor, and it is estimated by the subsequent equation [34]:

(17) $$k_{v}=0.0144-2.16\times 10^{-5}{(T}_{m}+273)+1.32\times 10^{-7}{\left(T_{m}+273\right)}^{2}$$

### 3.2. Boundary Conditions

The boundary conditions used with the mass, momentum, and energy transport equations are listed in the following tables.

#### 3.2.1. Mass Transport

The boundary conditions for the mass transport equation, applied to both the feed and membrane domains, are summarized in Table 2.

Here, $c_{h}$ represents water vapor concentration at the feed–membrane interface, and $c_{c}$ denotes water vapor concentration at the membrane–water gap interface. These values correspond to the saturation state of water vapor on both sides of membrane, driven by feed water evaporation at the feed side and vapor condensation at the water gap side. The saturation pressures associated with these concentrations are determined using the Antoine equation [35] as follows:

(18) $$P_{sat,f}=133.416x_{w}a_{w}\times 10^{8.10765-\frac{1750.286}{T_{m}+235}}$$

(19) $$P_{sat,g}=133.416\times 10^{8.10765-\frac{1750.286}{T_{m}+235}}$$

Here, $P_{sat,f}$ and $P_{sat,g}$ represent the saturation pressures at the feed–membrane and membrane–water gap interfaces, respectively. $x_{w}$ represents the water mole fraction, and $a_{w}$ refers to the water activity coefficient. Both $x_{w}$ and $a_{x}$ are determined as follows [34]:

(20) $$x_{w}=1-x_{NaCl}$$

(21) $$a_{w}=1-0.5x_{NaCl}-10x_{NaCl}^{2}$$

where $x_{NaCl}$ is the mole fraction of salt in the feed solution.

The vapor concentrations at feed–membrane and membrane–water gap interfaces are derived from the corresponding saturation vapor pressures, as in the following equations:

(22) $$c_{sat}=\frac{W}{v_{v}M_{w}}$$

(23) $$W=0.621945\frac{P_{sat}}{P_{atm}-P_{sat}}$$

(24) $$v_{v}=0.28704{(T}_{m}+273)\frac{1+1.607858W}{10^{-3}P_{atm}}$$

where $c_{sat}$ represents the saturation concentration of water vapor, $M_{w}$ is the molecular mass of water, $P_{atm}$ is the atmospheric pressure, $v_{v}$ is the specific volume of water vapor, $T_{m}$ is the local temperature at the membrane interfaces, and W denotes the vapor content.

#### 3.2.2. Momentum Transport

Table 3 outlines the boundary conditions employed for momentum transport physics across the feed, circulating water gap, and cooling channel domains.

#### 3.2.3. Energy Transport

The energy equation, for all domains, is solved by considering the boundary conditions presented in Table 4.

During the evaporation of water at the feed–membrane interface, latent heat is absorbed from the feed water. On the opposite side of the membrane, the vapor condenses, releasing heat into the water gap. To model this thermal behavior, a heat sink boundary condition is applied at the feed–membrane interface, whereas a heat source boundary condition is applied at the membrane–water interface. The corresponding heat values is determined as follows:

(25) $$q=\frac{J}{3600}h_{fg}$$

where J is the diffusive water flux, and $h_{fg}$ is the latent heat of vaporization or condensation, which can be determined using the following equation:

(26) $$h_{fg}=(2494-2.2\;T_{m})\times 10^{3}$$

where $T_{m}$ is the membrane local temperature for both membrane interfaces.

### 3.3. Specific Thermal Energy Consumption

To assess the thermal performance of the desalination module, STEC is employed as a key indicator. STEC represents the amount of thermal energy consumed by the desalination system per unit mass of produced permeate and is expressed in kWh/m^3^. It is determined using the following equation:

$$STEC=\frac{{\dot{m}}_{f}C_{p_{f}}({T_{f}}_{i}-T_{c_{o}})}{JA_{m}}$$

where ${\dot{m}}_{f}$ denotes the feed mass flow rate in kg/s, $C_{p_{f}}$ represents the specific heat capacity of the feed water, $T_{fi}$ and $T_{co}$ correspond to the feed inlet and coolant outlet temperatures, respectively, J refers to the water flux, and $A_{m}$ is the membrane surface area in m^2^.

### 3.4. Solving Technique

The finite element approach built in COMSOL Multiphysics is used to solve the current mathematical model. The transport of mass, momentum, and heat across the five domains depicted in Figure 3 is solved by means of COMSOL’s built-in modules. User-defined options and variables are used to integrate the rest of the expressions (Equations (2), (6)–(10), (18) and (19)).

The entire mathematical model is solved instantaneously across all domains as a totally coupled system. Throughout the analysis, the salt concentration is eliminated from the water gap space and considered to be 35,000 ppm at the inlet of the feed channel. As a result, the concentration convection–diffusion balance is used to model the distribution of water and salt concentrations in the feed channel.

### 3.5. Study on Number of Grid Elements

**Figure 4:**
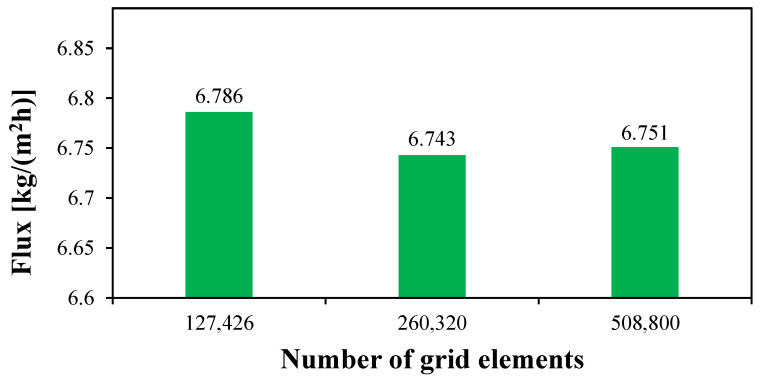
The grid independence study of the CFD simulation model for a circulating (${Re}_{g}=2712$) HF-WGMD module of ${Re}_{f}=2760$, ${Re}_{c}=146,$ and $T_{fi}=50\;{}^{\circ}C$.

The gird independence test is performed on a circulating HF-WGMD module of the length of 100 mm with a Reynolds number of 2760, 146, and 2712 in the feed, coolant, and water gap, respectively. The feed inlet temperature is kept at 50 °C with a salinity of 35,000 ppm. Three different grid levels (127,426, 260,320, and 508,800 elements) were used to test the CFD in this study. As illustrated in Figure 4, using 260,320 grid elements is excessively satisfactory, at which the produced water flux deviates only by 0.12% when doubling the number of grid elements to 508,800. Thus, driving the numerical analysis using 260,320 number of grid elements can save the computational time with a low level of discretization error.

## 4. Results and Discussion

### 4.1. Experimental Validation

The experimental validation of the CFD simulation is achieved by comparing the water flux from a stagnant HF-WGMD module, as proposed by Gao et al. [36], with the numerical flux produced by the same module specifications and operating conditions. The experimental module consisted of eight hollow fibers centered inside eight stainless steel tubes with 4.55 and 6.35 mm inner and outer diameters. The feed water was pumped through the lumen side of the hollow fibers of 0.8 and 1.16 mm inner and outer diameters at a 0.69 m/s inlet velocity and a 10,000 ppm inlet salinity. Meanwhile, the cooling water was permitted to pass through the module shell side at a 0.012 m/s inlet velocity.

**Figure 5:**
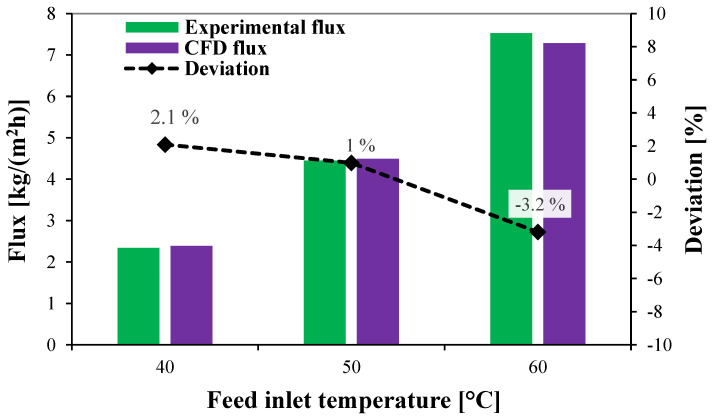
Experimental validation of the CFD simulation model using experimental data from reference [36].

As illustrated in Figure 5, three test cases are considered to validate the current CFD simulation model at different feed inlet temperatures of 40, 50, and 60 °C. The numerical water fluxes show excellent agreement with the experimental ones in all test cases. The maximum deviation is encountered at a 60 °C feed inlet temperature, at which the percentage deviation of −3.2% is recorded.

The current CFD simulation was previously validated using the experimental data of an HF-WGMD module that uses high-density polyethylene cooling tubes instead of stainless steel ones by Elbessomy et al. [15]. Specifically, the influence of feed and coolant inlet flow velocities was validated across a broad range. The study explored feed velocities from 0.28 m/s to 0.81 m/s and coolant velocities from 0.003 m/s to 0.008 m/s, while a constant feed inlet temperature of 70 °C was maintained. The numerical predictions of water flux demonstrated excellent agreement with the experimental measurements across all test cases. The maximum deviation between simulation and experimental data was found to be 4.2%, occurring at feed and coolant inlet velocities of 0.4 m/s and 0.004 m/s, respectively.

### 4.2. CFD Simulations

In the following sections, the CFD simulations are employed to generate temperature contour plots, which offer a visual representation of the temperature distributions within the various domains of the 100 mm HF-WGMD module operating at a feed inlet salinity of 35,000 ppm. This allows for a comprehensive evaluation of the influence of varying operational conditions on the module’s performance. Both laminar and turbulent flow regimes within the feed and cooling channels are analyzed to characterize the temperature polarization across the module. Additionally, the influence of the water gap circulation flow pattern on the temperature distribution within the water gap is examined, highlighting the enhancements facilitated by the circulation in the HF-WGMD module. The temperature gradient across both the membrane and water gap is investigated under different feed inlet temperatures, comparing scenarios with stagnant and circulating flow conditions within the HF-WGMD module. For all cases, a section 3 mm in length at the mid-point of the module is considered for evaluation.

#### 4.2.1. Feed Water Temperature Distribution at Different Feed Inlet Reynolds Numbers of Stagnant HF-WGMD Module

**Figure 6:**
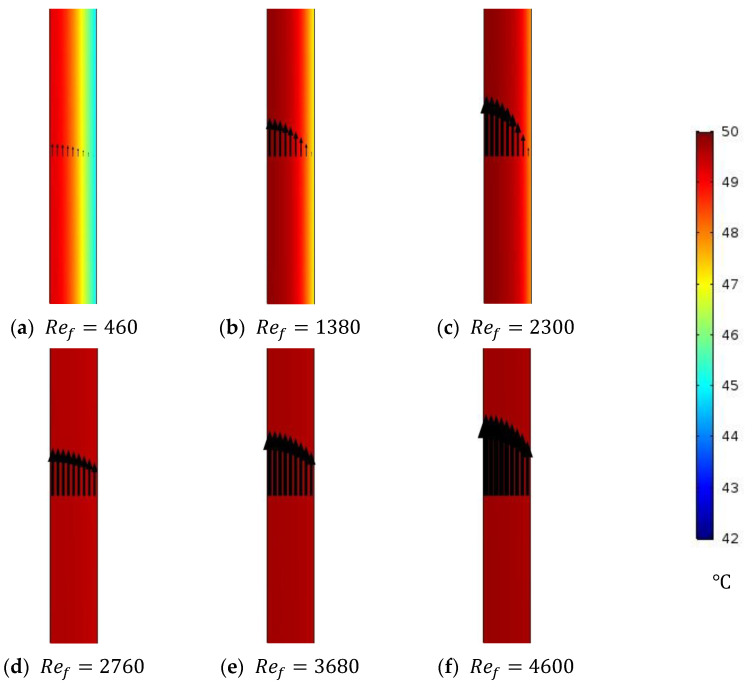
Temperature distribution of the feed channel of the stagnant HF-WGMD module for the different feed Reynolds numbers at $T_{fi}=50\;{}^{\circ}C$ and ${Re}_{c}=2300$.

The effect of feed water inlet velocities on the feed water temperature polarization is investigated and reported by illustrating the temperature contours inside the feed channel domain of a stagnant HF-WGMD module. The CFD is used to simulate the performance of a stagnant HF-WGMD module at different laminar and turbulent feed inlet Reynolds numbers, considering a constant feed inlet temperature of 50 °C and a coolant inlet Reynolds number of 2300. Figure 6 shows the temperature distribution of feed water for laminar feed flow patterns of 460, 1380, and 2300 Reynolds numbers and turbulent flow patterns of 2760, 3680, and 4600 Reynolds numbers. As it is obvious in the figure, the feed water temperature distribution enhances with the increasing flow Reynolds number in the case of laminar flow patterns due to the enhancement that occurs in the heat transfer mechanism. Uniform temperature distribution across the feed channel was observed at the three turbulent Reynolds numbers due to the induced turbulence and mixing of the feed water around the fibers. This shows the superiority to turbulent flow by reducing temperature polarization in the feed channel; hence, the module water flux can be enhanced.

Table 5 gives data for the average temperature and vapor concentration at the feed–membrane interface for different laminar and turbulent feed Reynolds numbers. As discussed, increasing the feed inlet Reynolds number significantly reduces temperature polarization at the feed–membrane interface. At this point, interface temperature enhances from 45.1 °C, at Reynolds number of 460, to 47.2 and 47.9 °C, at Reynolds numbers of 1380 and 2300, respectively. This induces water vapor concentration of 3.53, 3.9, and 4.04 mol/m^3^ at the corresponding Reynolds numbers, respectively. On the other side, higher temperatures are obtained in the turbulent feed flow cases with nearly no change. At this point, the interface temperature changes from 49.5 to 49.7 °C when the Reynolds number is increased from 2760 to 4600; hence, the average concentration remains almost constant at 4.4 mol/m^3^, as shown in Table 5.

#### 4.2.2. Water Gap, Cooling Tube, and Cooling Water Temperature Distributions at Different Coolant Inlet Reynolds Numbers of Stagnant HF-WGMD Module

**Figure 7:**
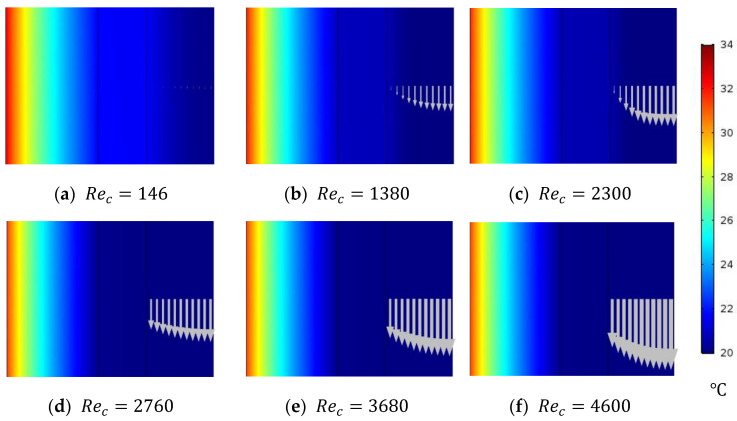
Temperature distribution of WG, cooling tube, and cooling water through the stagnant HF-WGMD module for different coolant Reynolds numbers at $T_{fi}=50\;{}^{\circ}C$ and ${Re}_{f}=2300$.

The major function of the cooling water in the WGMD configuration is to provide sufficient cooling to the water gap. Thus, the CFD simulation is used to investigate the effect of cooling water Reynolds number on the WG temperature. The study is conducted on a stagnant HF-WGMD module at a 50 °C feed inlet temperature while keeping 2300 as the feed inlet Reynolds number. Figure 7 shows the temperature distribution inside the WG, cooling tube, and cooling water domains at different laminar (146, 1380 and 2300) and turbulent (2760, 3680 and 4600) Reynolds numbers. It is noticed that the coolant Reynolds number has a negligible effect on the WG temperature for all laminar flow cases. Slightly lower temperatures were observed for the water guide and cooling tube at turbulent flow patterns. Hence, it can be deduced that the hydraulic specifications of the cooling channel have minimal effects on the HF-WGMD module water flux.

**Figure 8:**
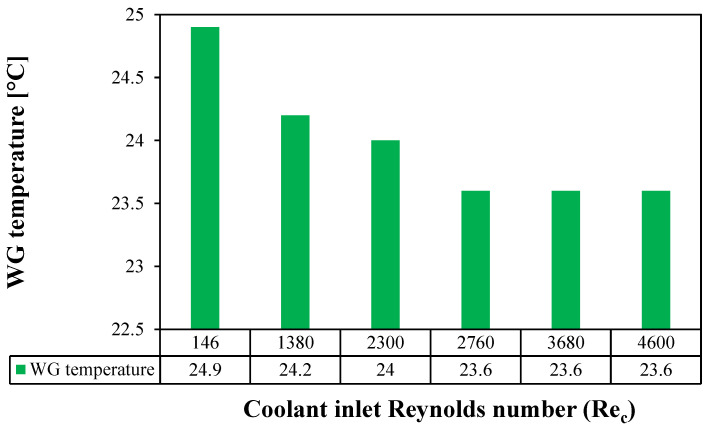
Average WG temperature of the stagnant HF-WGMD module versus the coolant Reynolds number at $T_{fi}=50\;{}^{\circ}C$ and ${Re}_{f}=2300$.

Figure 8 illustrates the average temperature of the WG of the HF-WGMD module against the cooling water Reynolds number. Increasing the cooling water laminar Reynolds number from 146 to 2300 decreases the WG temperature from 24.9 to 24 °C, representing only 3.6% of the enhancement in WG temperature. WG temperature decreases to 23.6 °C when applying turbulent flow patterns to the cooling water, providing a maximum WG temperature reduction of 5.2% lower than that at a Reynolds number of 146.

#### 4.2.3. WG Temperature Distribution at Different WG Circulation Reynolds Numbers of Circulating HF-WGMD Module

**Figure 9:**
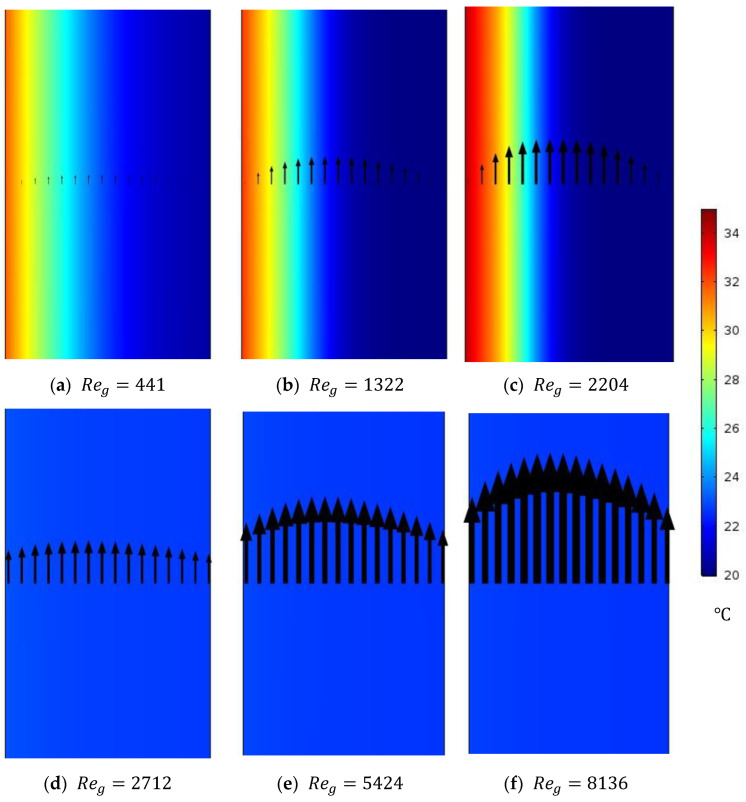
Temperature distribution of WG through the circulating HF-WGMD module for different WG circulation Reynolds numbers at $T_{fi}=50\;{}^{\circ}C$, ${Re}_{f}=2760,$ and ${Re}_{c}=146$.

In contrast to the trials to increase the feed and coolant velocities in the previous two sections, the effect of circulating the distillate water on the WG temperature distribution to assess the cooling pattern of membrane–WG interface at different WG Reynolds numbers will be presented in this section. Figure 9 illustrates the temperature contours inside the WG domain of circulating HF-WGMD module at different laminar and turbulent WG Reynolds numbers. The study is conducted on the circulating HF-WGMD module at a 50 °C feed inlet temperature while keeping the feed and coolant inlet Reynolds numbers at 2760 and 146, respectively. Although a higher Reynolds number improves heat transfer in the radial direction within the water gap, the laminar flow nature prevents this heat from reaching the coolant side. As a result, heat accumulates near the membrane–water gap interface, elevating the local temperature at this interface, as clearly shown when comparing the CFD simulations in Figure 9a–c. On the other side, increasing the WG Reynolds number to turbulent flow levels (${Re}_{g}>2300$) enhances the internal mixing between the flow layers, providing better cooling to the membrane–WG interface. No change in the WG temperature distribution is observed in all turbulent flow cases, as illustrated in Figure 9d–f.

**Figure 10:**
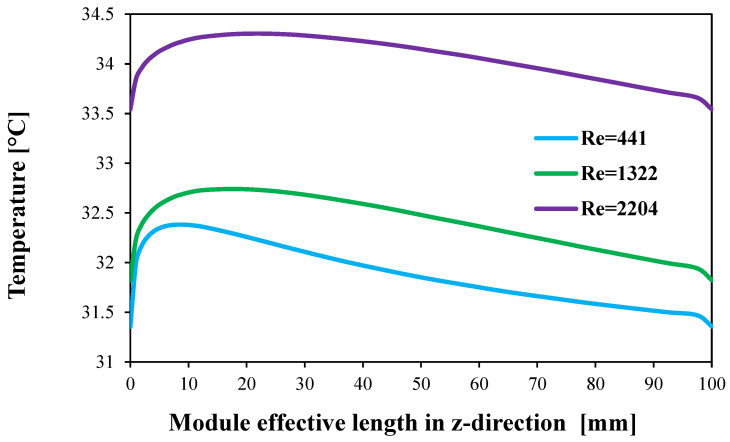
Temperature profile along membrane–WG interface for different WG circulation Reynolds numbers at $T_{fi}=50\;{}^{\circ}C$, ${Re}_{f}=2760$, and ${Re}_{c}=146$.

The temperature profile at the membrane–water gap interface for a single-stage HF-WGMD module (100 mm in length) is presented in Figure 10. The results show that, within the laminar regime, increasing the Reynolds number leads to a higher localized temperature at the membrane–water gap interface. For a given Reynolds number, the localized temperature rises sharply near the entrance region, then gradually decreases until it reaches the same value at the water gap outlet. This behavior is influenced by the flow arrangement: the hot feed flows in parallel with the circulating water, while the coolant moves in a counterflow direction.

Table 6 gives average temperatures and vapor concentrations at the membrane–WG interface at different laminar and turbulent flow Reynolds numbers. Increasing laminar WG Reynolds number from 441 to 2204 increases the membrane–WG interface temperature from 31.9 to 34.1 °C which negatively elevates water vapor concentrations from 1.88 to 2.11 mol/m^3^, while increasing WG Reynolds number to turbulent flow levels (2712, 5424, and 8136) reduces the average temperature to nearly 23 °C in all turbulent cases, providing 27.9% decrease in temperature than at a WG Reynolds number of 441. Hence, the water vapor concentration at the membrane–WG interface positively decreases to about 1.15 mol/m^3^ in all turbulent cases providing a water vapor concentration reduction of 38.8% compared to that for a WG Reynolds number of 441. This shows the superiority of turbulent WG circulation regime in providing a better cooling pattern for the membrane of the HF-WGMD module.

#### 4.2.4. Membrane and WG Temperature Distribution at Different Feed Inlet Temperatures of Stagnant and Circulating HF-WGMD Modules

**Figure 11:**
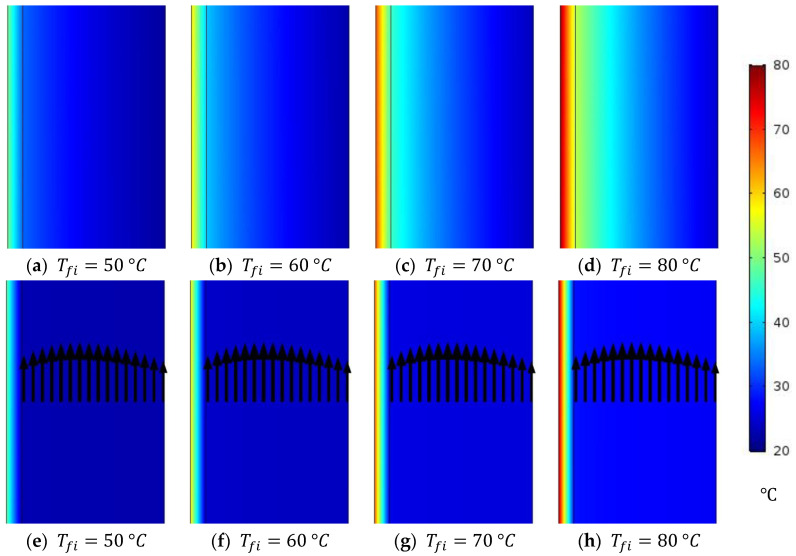
Temperature distribution of membrane and WG at different feed inlet temperatures through (**a**–**d**) stagnant and (**e**–**h**) circulating (${Re}_{g}=2712$) HF-WGMD modules at ${Re}_{f}=2760$ and ${Re}_{c}=146$.

CFD simulations were performed to evaluate the performance of both stagnant and circulating HF-WGMD modules at feed inlet temperatures of 50, 60, 70, and 80 °C. In both configurations, the feed and coolant inlet Reynolds numbers were maintained at 2760 and 146, respectively, while the circulating module was operated at a WG Reynolds number of 2712. Figure 11 shows the temperature distribution through the membrane layer and WG for both configurations. An increase in the feed inlet temperature leads to an elevated feed–membrane interface temperature, which in turn increases the water vapor concentration based on the Antoine equation (Equation (18)). In the circulating configuration, the introduction of distillate water circulation enhances the mixing between the WG layers, reducing the temperature polarization effect near the membrane–WG interface. This is clearly illustrated by the lower temperatures at the membrane cold side in the circulating configuration compared to the stagnant one.

**Figure 12:**
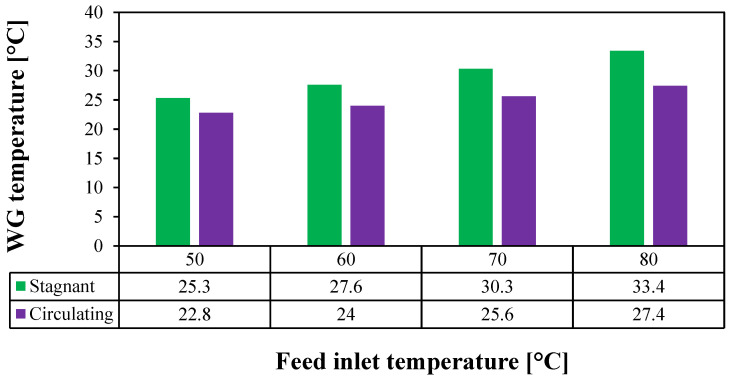
Average WG temperature of the stagnant and circulating (${Re}_{g}=2712$) HF-WGMD modules versus the feed inlet temperature at ${Re}_{f}=2760$ and ${Re}_{c}=146$.

Additionally, Figure 12 compares the average WG temperature between the two configurations. At a 50 °C feed inlet temperature, the WG temperature decreases from 25.3 °C in the stagnant module to 22.8 °C in the circulating module, representing a 9.9% reduction. Higher feed inlet temperatures yield greater reductions, with WG temperature decreases of 13%, 15.5%, and 18% at 60, 70, and 80 °C, respectively. These findings demonstrate that the enhanced mixing due to circulation significantly mitigates temperature polarization, thereby improving the overall performance of the HF-WGMD module.

### 4.3. Parametric Study on Single Stage of Stagnant and Circulating HF-WGMD Modules

The impact of varying operating conditions on the productivity of the HF-WGMD module is examined systematically to identify the best performance parameters. A 100 mm HF-WGMD module, configured with both stagnant and circulating water gaps, is analyzed across a broad range of laminar and turbulent Reynolds numbers, while maintaining fixed inlet feed temperature and salinity conditions of 80 °C and 35,000 ppm, respectively. In addition, the performance of the circulating HF-WGMD module is evaluated at different feed inlet temperatures (50, 60, 70, and 80 °C) and compared to the performance of the conventional stagnant HF-WGMD module under the same feed and coolant Reynolds number conditions.

#### 4.3.1. Effect of Feed Flow Reynolds Number on Water Flux of Stagnant HF-WGMD Module

**Figure 13:**
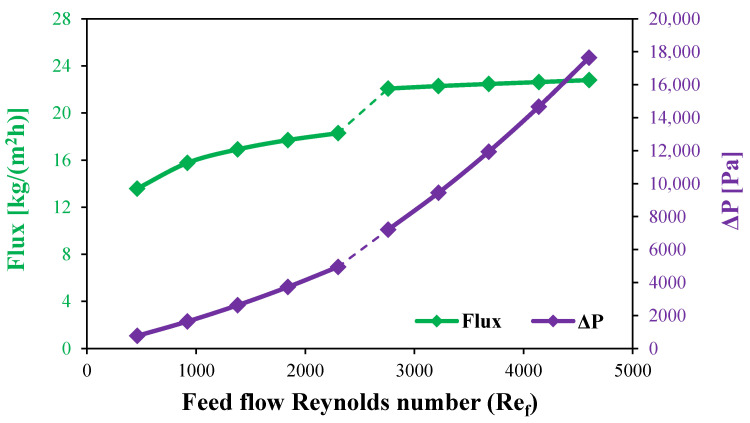
Water flux and feed water pressure drop for stagnant HF-WGMD module versus feed Reynolds number at ${Re}_{c}=2300$.

This section examines the distillate water flux from a stagnant HF-WGMD module under varying laminar and turbulent feed inlet Reynolds numbers. Figure 13 illustrates the relationship between the water flux and the feed inlet Reynolds number at a fixed feed inlet temperature of 80 °C and a coolant inlet temperature of 20 °C. At a feed inlet Reynolds number of 460, the water flux is measured at 13.58 kg/(m^2^h). As the Reynolds number increases to 1380 and 2300, the water flux correspondingly rises to 16.91 and 18.29 kg/(m^2^h), representing water flux enhancements of 24.5% and 34.7%, respectively. This increase in water flux with a rising Reynolds number in the laminar regime is attributed to improved convective transport, which mitigates temperature polarization effects at the membrane interface.

Furthermore, the transition from laminar to turbulent flow markedly enhances the water flux. Specifically, the water flux increases from 18.29 kg/(m^2^h) at a Reynolds number of 2300 (laminar regime) to 22.08 kg/(m^2^h) at a Reynolds number of 2760, achieving an additional enhancement of 20.7%. Beyond this point, further increases in turbulent Reynolds number yield diminishing returns; for instance, raising the feed Reynolds number from 2760 to 4600 results in only a 3.2% increase in water flux, indicating that the system reaches a near-saturation point in performance improvements under the studied conditions, as illustrated in Figure 13.

Overall, these observations underscore the significant impact of flow regime and Reynolds number on the performance of the HF-WGMD module, particularly in balancing enhanced convective mass transfer with the onset of turbulent flow dynamics.

Figure 13 illustrates the effect of increasing the feed inlet Reynolds number on the pressure drop along the feed channel. As the feed flow remains within the laminar regime, increasing the Reynolds number from 460 to 2300 results in a substantial rise in the pressure drop from 765.5 to 4948.9 Pa. This increase is attributed to the enhanced velocity gradient near the channel walls. Upon reaching the transition regime, where the Reynolds number increases from 2300 to 2760, the pressure drop experiences a further escalation from 4948.9 to 7205.9 Pa, marking a 45.6% increase. This is primarily due to the onset of turbulent eddies and fluctuations, which increase energy dissipation and resistance to fluid motion.

In contrast, operating under fully turbulent conditions leads to a pronounced increase in the pressure drop. When the Reynolds number rises from 2760 to 4600, the pressure drop sharply increases to 17,640 Pa, representing a significant 144.8% increase. This drastic rise is primarily caused by the intensified turbulence, which amplifies flow resistance. However, despite the substantial increase in the pressure drop at high turbulent flow conditions, the corresponding gain in water flux is found to be negligible, as depicted in Figure 13. This suggests that, beyond a certain threshold, further increasing the Reynolds number results in excessive pressure losses without a proportional improvement in system productivity.

#### 4.3.2. Effect of Coolant Flow Reynolds Number on Water Flux of Stagnant HF-WGMD Module

**Figure 14:**
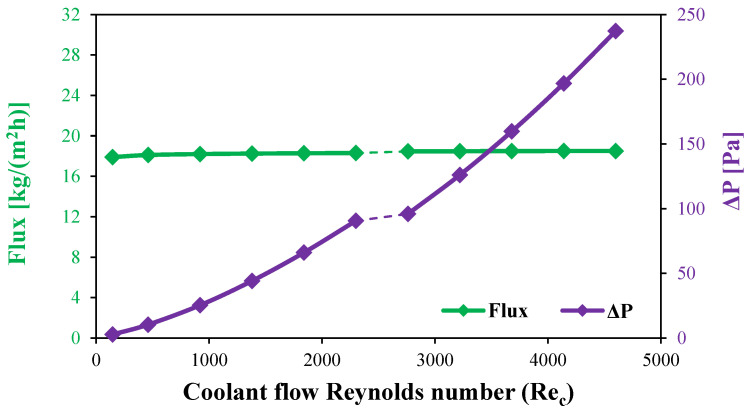
Water flux and cooling water pressure drop for stagnant HF-WGMD module versus coolant Reynolds number at ${Re}_{f}=2300$.

The water flux of stagnant HF-WGMD module is studied versus the cooling water inlet Reynolds number. The investigation is conducted on a stagnant HF-WGMD module at an 80 °C feed inlet temperature while keeping the coolant inlet at 20 °C. In the stagnant HF-WGMD configuration, the cooling channel is separated from the membrane by a stagnant water gap and the cooling tube. The higher thermal resistance of this stagnant water gap reduces the influence of coolant flow characteristics on the performance of the WGMD module. Therefore, no significant enhancement in water flux is observed with increasing the coolant inlet Reynolds number in both laminar and turbulent flow regimes, as depicted in Figure 14. At this point, HF-WGMD module produces 17.9 kg/(m^2^h) of water flux at a coolant inlet Reynolds number of 146, and the water flux slightly increases to 18.25 and 18.3 kg/(m^2^h) when dealing with higher laminar Reynolds numbers of 1380 and 2300. Additionally, transition from a laminar to a turbulent coolant flow pattern shows no more enhancement in flux at which 18.46 kg/(m^2^h) of water flux is observed at all turbulent Reynolds numbers.

Moreover, Figure 14 presents the variation in pressure drop along the cooling water channel under both laminar and turbulent flow conditions. At a coolant Reynolds number of 146, the pressure drop is relatively minimal, reaching only 2.7 Pa. However, as the Reynolds number increases, a substantial rise in the pressure drop is observed. Specifically, at a Reynolds number of 2300 (the upper limit of the laminar regime), the pressure drop escalates to 90.6 Pa. This increase becomes even more pronounced in the turbulent regime, where the pressure drop reaches 237.2 Pa at a Reynolds number of 4600.

Notably, despite this significant increase in pressure losses, no corresponding enhancement in water flux is observed, as depicted in Figure 14. This indicates that operating at higher Reynolds numbers in the cooling channel leads to excessive energy dissipation without improving system performance. Consequently, a coolant Reynolds number of 146 appears to be sufficient for maintaining optimal water flux while minimizing pressure losses, making it the most efficient choice for system operation.

#### 4.3.3. Effect of WG Circulation Reynolds Number on Water Flux of Circulating HF-WGMD Module at Different Feed Inlet Temperatures

**Figure 15:**
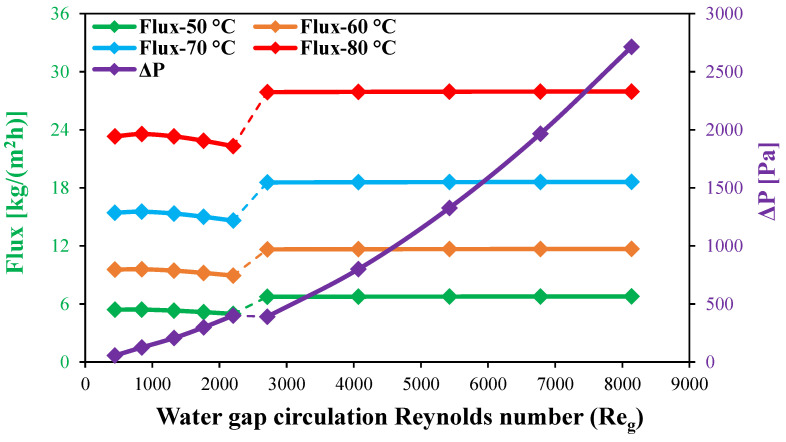
Water flux and WG pressure drop for circulating HF-WGMD module versus WG circulation Reynolds number at ${Re}_{f}=2760$, ${Re}_{c}=146,$ and different feed inlet temperatures.

In this section, the effect of WG circulation configuration on the module water flux is investigated for a wide range of laminar and turbulent circulation Reynolds numbers at different feed inlet temperatures. Various laminar and turbulent WG Reynolds numbers are tested while keeping the feed and coolant inlet Reynolds numbers at 2760 and 146, respectively. As discussed in Section 4.2.3, the elevated local temperature at the membrane–water gap interface at higher Reynolds numbers within the laminar flow regime results in accumulated heat that is not transferred to the coolant side, thereby reducing the water flux, as shown in Figure 15. The primary purpose of the water gap is to minimize heat conduction losses through the membrane while dissipating the latent heat of condensation from the permeate to the coolant side. However, in the stagnant water gap configuration, water flux is inherently low, and increasing the Reynolds number within the laminar regime further reduces water flux, as demonstrated in this study. Therefore, the current study reveals a declining trend in water flux with an increase in the WG Reynolds number in the case of laminar flow regime (${Re}_{g}<2300$), as illustrated in Figure 15. The water flux decreases from 23.31 kg/(m^2^h) at a WG Reynolds number of 441 to 22.31 kg/(m^2^h) with an increase in WG Reynolds number to 2204, representing 4.3% reduction in flux at an 80 °C feed inlet temperature. While the water flux is 5.41, 9.55, and 15.43 kg/(m^2^h) for a WG Reynolds number of 441, it decreases by 8.1%, 6.5%, and 5.3% with an increase in the WG Reynolds number to 2204 at feed inlet temperatures of 50, 60, and 70 °C, respectively.

On the other side, a significant increase in the module water flux is observed with the transition from a laminar to a turbulent flow pattern as a result of the enhancement in WG flow characteristics at all feed inlet temperatures. The HF-WGMD module produces 27.89 kg/(m^2^h) of water flux at a WG Reynolds number of 2712, producing up to 19.6% of enhancement in water flux over that produced in the case of a WG Reynolds number of 441 (the best laminar case) at an 80 °C feed inlet temperature, as presented in Figure 15. Higher percentages of enhancements of 24.6%, 21.9, and 20.3% are produced at feed inlet temperatures of 50, 60, and 70 °C, respectively. At higher Reynolds numbers, no more increase in water flux is observed due to unchanged flow characteristics, as discussed in Section 4.2.3.

Figure 15 illustrates the variation in WG pressure drop along the WG channel, providing insights into the effectiveness of different WG circulation configurations. The results indicate a substantial increase in the pressure drop from 55.8 Pa to 400 Pa as the WG Reynolds number increases within the laminar regime from 441 to 2204, coinciding with a decline in water flux.

Upon transitioning from laminar (${Re}_{g}=2204$) to turbulent flow (${Re}_{g}=2712$), the pressure drop exhibits a minor reduction to 390.4 Pa due to improved flow characteristics. However, at higher turbulent Reynolds numbers, a sharp escalation in the pressure drop is observed, reaching a peak of 2713.3 Pa at a WG Reynolds number of 8136. This significant increase is attributed to intensified frictional losses associated with high-velocity turbulent flow.

Based on the trade-off between the water flux enhancement and pressure drop, the best WG circulation configuration in this study is determined to be at ${Re}_{g}=2712$. Under this condition, the pressure drop per unit water flux is 57.9, 33.5, 21, and 14 Pa/kg/(m^2^h), which represents a reduction of approximately 28.1%, 25.2%, 23.3%, and 21.9% compared to ${Re}_{g}=2204$ at feed inlet temperatures of 50, 60, 70, and 80 °C, respectively.

#### 4.3.4. Effect of Feed Water Inlet Temperature on Water Flux of Stagnant and Circulating HF-WGMD Modules

**Figure 16:**
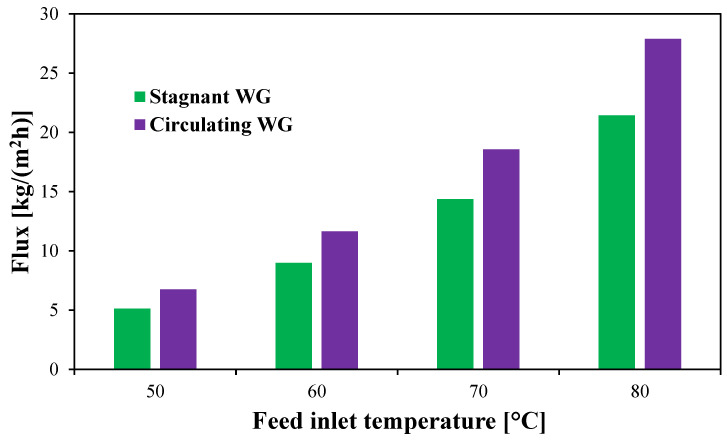
Water flux of stagnant and circulating (${Re}_{g}=2712$) HF-WGMD module versus feed inlet temperature at ${Re}_{f}=2760$ and ${Re}_{c}=146$.

A comparative study is performed using the CFD simulations to assess the effect of the WG circulation configuration on water flux compared to the conventional stagnant HF-WGMD configuration at different feed inlet temperatures. Herein, the HF-WGMD module is simulated with feed and coolant inlet Reynolds numbers of 2760 and 146, respectively, with both stagnant and circulating water gap configurations. In the case of circulating HF-WGMD module only, WG is circulated with Reynolds number of 2712. In general, the module productivity is enhanced with an increase in the feed water temperature due to the enhancement in water vapor concentration at the feed–membrane interface, as depicted in Antione equation (Equation (18)). The water flux produced by the stagnant HF-WGMD module is increased from 5.13 kg/(m^2^h) at a feed inlet temperature of 50 °C to 8.99, 14.38, and 21.43 kg/(m^2^h) at feed inlet temperatures of 60, 70, and 80 °C, respectively, as illustrated in Figure 16.

Moreover, the water flux is enhanced using the circulating WG configuration, as shown in Figure 16. At a feed inlet temperature of 50 °C, the flux is increased to 6.74 kg/(m^2^h), yielding about 31.4% of distillate water over that produced by the stagnant HF-WGMD module at the same feed inlet conditions. Slightly lower percentages of enhancements are observed at higher feed temperatures. At this point, the flux is enhanced by 29.5%, 29.1%, and 30.1% at feed inlet temperatures of 60, 70, and 80 °C, respectively, when using the circulating HF-WGMD module instead of the stagnant HF-WGMD module.

### 4.4. Performance Assessment of Stagnant and Circulating Multistage HF-WGMD Modules

To evaluate the scalability and practical applicability of both stagnant and circulating HF-WGMD modules for potential use in large-scale desalination plants, this study conducts a detailed investigation covering systems with up to 50 stages. The analysis examines how each configuration performs in terms of water production, thermal efficiency, and operational considerations across varying stage numbers. This approach provides valuable insights into the trade-offs between the two designs and identifies the conditions under which each configuration is most advantageous for large-scale implementation. This section evaluates the performance of multistage modules in terms of productivity and energy consumption at different feed inlet temperatures (50, 60, 70, and 80 °C). The coolant inlet temperature is fixed at 20 °C. Reynolds numbers for the feed, coolant, and circulating water gap are fixed at 2760, 146, and 2712, respectively. The feed inlet salinity remains constant at 35,000 ppm. The study investigates the productivity and thermal performance of the HF-WGMD modules connected in series, considering up to 50 stages.

#### 4.4.1. Water Flux and Energy Recovered

**Figure 17:**
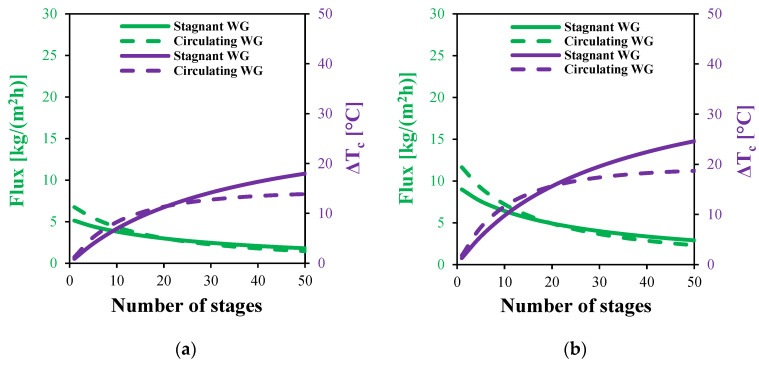

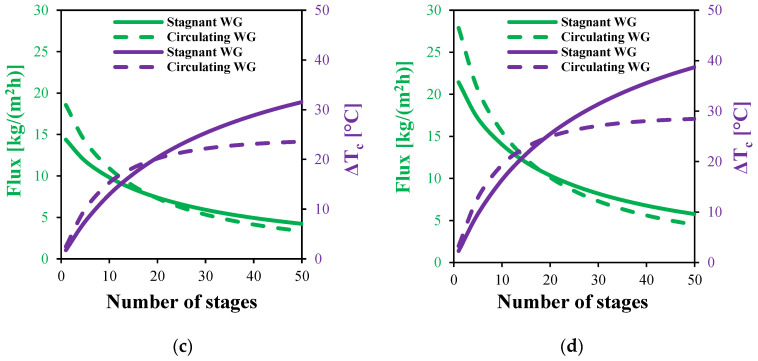
The effect of the number of stages on the water flux and cooling water temperature rise of MS-HF-WGMD systems at different feed inlet temperatures of (**a**) 50 °C, (**b**) 60 °C, (**c**) 70 °C, and (**d**) 80 °C.

The variations in water flux with the number of stages for both the stagnant and circulating HF-WGMD configurations at different feed inlet temperatures are presented in Figure 17. The results reveal a general decline in the water flux as the number of stages increases for both configurations. This decrease is primarily due to the intensification of temperature and concentration polarization effects while increasing the overall module length, which reduces the driving concentration difference across the membrane.

At a feed inlet temperature of 80 °C, the circulating HF-WGMD module demonstrates an initial performance advantage, achieving a water flux of 27.89 kg/(m^2^h) in a single stage system, which is 30.1% higher than the water flux of 21.4 kg/(m^2^h) observed in the stagnant configuration. This enhancement is primarily due to improved mixing and convective mass transfer within the water gap. However, this advantage diminishes with additional stages; by the 15th stage, the water flux difference narrows to 3.5%, and beyond 20 stages, the stagnant module begins to outperform its circulating counterpart. At 50 stages, the stagnant module achieves a 21.9% higher flux (5.76 against 4.5 kg/(m^2^h)). This trend persists across all tested feed temperatures. For single stage systems at 50, 60, and 70 °C, the circulating module achieves water fluxes of 6.7, 11.6, and 18.6 kg/(m^2^h), outperforming the stagnant module’s 5.1, 9.0, and 14.4 kg/(m^2^h), respectively. Conversely, at 50 stages, the stagnant module exhibits superior water fluxes of 1.8, 2.9, and 4.2 kg/(m^2^h), compared to 1.5, 2.3, and 3.3 kg/(m^2^h) for the circulating module at the same temperatures.

Furthermore, Figure 17 illustrates the cooling water temperature rise, an indicator of thermal energy recovery, as a function of the number of stages. For stage counts below 20, both the circulating and stagnant configurations exhibit an increasing temperature rise, with the circulating module showing higher values and a steeper growth rate. At a feed inlet temperature of 80 °C and five stages, the circulating module achieves a temperature rise of 12.85 °C, 33.4% higher than the stagnant module’s 9.63 °C. However, this advantage diminishes with additional stages; by 20 stages, both configurations converge at approximately 25 °C. Beyond this point, the temperature rise in the circulating module plateaus around 28 °C, while the stagnant module continues to increase, ultimately surpassing the circulating configuration. At 50 stages and 80 °C, the stagnant module records a 36% higher temperature rise. This behavior is consistent across lower feed temperatures. For instance, at 50 stages, the stagnant module outperforms the circulating module by 29.5%, 31.6%, and 33.7% at feed temperatures of 50, 70, and 80 °C, respectively, confirming that the stagnant configuration becomes more thermally efficient at high stage counts.

#### 4.4.2. Specific Thermal Energy Consumption

**Figure 18:**
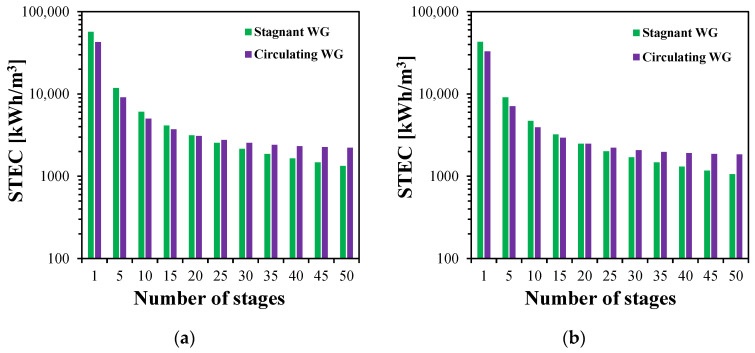

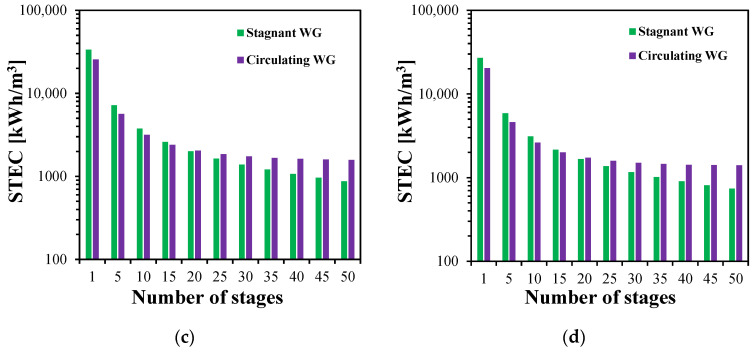
The effect of the number of stages on the STEC of MS-HF-WGMD systems at different feed inlet temperatures of (**a**) 50 °C, (**b**) 60 °C, (**c**) 70 °C, and (**d**) 80 °C.

The STEC of the stagnant and circulating MS-HF-WGMD systems is analyzed and presented for different numbers of stages and different feed inlet temperatures, as shown in Figure 18. In both configurations, the STEC decreases with the increase in the number of stages, primarily due to enhanced system productivity from an extended module length and improved thermal energy recovery by the coolant, which reduces the energy required to heat the feed stream. At 80 °C, the STEC in the circulating module drops from 20,349 to 1402 kWh/m^3^ as the number of stages increases from 1 to 50, while the stagnant module shows a decrease from 26,945 to 793 kWh/m^3^. For fewer than 20 stages, the circulating module exhibits a lower STEC than the stagnant one, indicating superior thermal performance. However, beyond 20 stages, the stagnant configuration achieves a more rapid reduction in the STEC. As shown in Figure 18d, at 50 stages, the stagnant module consumes approximately 47.3% less thermal energy than the circulating module to produce the same amount of distillate water. This crossover behavior is consistent across all tested feed temperatures (Figure 18a–c), highlighting the diminishing benefit of circulation with the increase in stage number.

## 5. Conclusions

A 2D axisymmetric CFD model is established to simulate the circulation of distillate water through the water gap of an HF-WGMD module. The proposed circulating HF-WGMD module performance is assessed and compared with that of the conventional HF-WGMD module with a stagnant water gap configuration at different feed temperatures. The impact of feed and coolant inlet Reynolds number on the MD module is investigated. The HF-WGMD module with a WG circulation configuration is tested for different laminar and turbulent circulation Reynolds numbers. In addition, a study on a multistage system level is implemented for both WG configurations. The following points succinctly describe the primary findings and future recommendations of the current study:

- Transitioning from a laminar to a turbulent feed flow regime assures up to 20.7% more water flux when a stagnant WG configuration at a feed inlet temperature of 80 °C is considered.
- Enhancing the heat transfer characteristics of the cooling channel—by increasing the Reynolds number of the coolant stream—had a negligible impact on the WG temperature. This limited influence is attributed to the dominant thermal resistance posed by the stagnant water gap, which is significantly higher than that of the cooling tube and channel.
- In the circulating HF-WGMD modules, increasing the WG Reynolds number within the laminar regime reduces water flux, while water flux remains stable across all turbulent flow conditions.
- Transitioning from laminar to turbulent WG circulation boosts productivity; at Re = 2712, the circulating HF-WGMD module achieves 27.89 kg/(m^2^h) water flux, representing a 19.6% higher water flux than at Re = 441, under an 80 °C feed temperature.
- At all feed inlet temperatures, utilizing a single stage of circulation WG configuration outperforms the stagnant WG configuration in terms of the water output flux, at which the water flux is enhanced by up to 31.4% at a 50 °C feed inlet temperature.
- At 80 °C, a 50-stage stagnant WG system consumes only 793 kWh/m^3^, representing a 47.3% reduction compared to circulating WG.
- For multistage HF-WGMD systems, modules with circulating WG outperform stagnant ones when the number of stages is below 20. However, beyond this threshold, the stagnant configuration achieves a higher water flux and a lower STEC, making it more suitable for scalability in large-scale water desalination plants.
- It is important to note that the present study, although it provides valuable insights, does not account for all energy consumption. A complete understanding of the pumping power needed for water gap circulation, which includes losses from fittings and pipes, would require a detailed design of the full-scale vessel and specific operational assumptions. This detailed analysis of parasitic energy losses is a complex but relevant topic that deserves to be a separate research topic in the future.
- Further investigations are recommended to examine the effects of additional geometric parameters (e.g., water gap thickness) and operational conditions (e.g., feed salinity) on the performance of the circulating HF-WGMD module.
- While membrane wetting is primarily influenced by factors such as the chemical composition and surface properties of the membrane material—including hydrophobicity, pore size, and surface roughness—as well as feed flow characteristics, the effect of changing the permeate-side flow configuration in this study requires further investigation. Future experiments for long-term operation under real conditions will be designed to investigate the conditions that cause wetting and how it affects water flux and rejection rates under different water gap configurations.

## Figures and Tables

**Table 1:** HF-WGMD module specifications.

Parameter	Symbol	Value	Unit
Membrane inner radius	$r_{f-m}$	0.4	mm
Membrane outer radius	$r_{m-g}$	0.58	mm
Cooling tube inner radius	$r_{g-t}$	2.28	mm
Cooling tube outer radius	$r_{t-c\;}$	3.18	mm
Coolant channel radius	$r_{c}$	4.42	mm
Module effective length	L	100	mm
Feed inlet salinity	−	35,000	ppm
Feed water thermal conductivity	$k_{f}$	0.64	W/(m K)
Membrane thermal conductivity	$k_{m}$	0.07	W/(m K)
Membrane porosity	ε	82	%
Membrane pore tortuosity	τ	1.7	-
Membrane pore diameter	$d_{P}$	0.16	μm
Water gap salinity	−	0.0	ppm
Cooling tube thermal conductivity	$k_{t}$	15	W/(m K)
Vapor molecular mass (H_2_O)	$M_{w}$	18	g/mol
Salt molecular mass (NaCl)	$M_{NaCl}$	58.5	g/mol

**Table 2:** Boundary conditions for the mass transport equations.

Domain	Position	Boundary Condition
Feed	r=0	$\frac{\partial c_{f}}{\partial r}=0$
	$r=r_{f-m}$	$-D_{w}\frac{\partial c_{f}}{\partial r}=-D_{m}\frac{\partial c_{m}}{\partial r}$
	z=0	$c_{f}=c_{fi}$
	z=L	$\frac{\partial c_{f}}{\partial z}=0$
Membrane	$r=r_{f-m}$	$c=c_{h}$
	$r=r_{m-g}$	$c=c_{c}$
	z=0	$\frac{\partial c_{m}}{\partial z}=0$
	z=L	$\frac{\partial c_{m}}{\partial z}=0$

**Table 3:** Boundary conditions for the momentum transport equations.

Domain	Position	Boundary Condition
Feed	r=0	$\frac{\partial u_{f}}{\partial r}=0,\;\frac{\partial w_{f}}{\partial r}=0$
	$r=r_{f-m}$	$u_{f}=0,w_{f}=0$
	z=0	$u_{f}=0,w_{f}=U_{fi}$
	z=L	$P_{f}=P_{atm}$
Circulating WG	$r=r_{m-g}$	$u_{g}=0,w_{g}=0$
	$r=r_{g-t}$	$u_{g}=0,w_{g}=0$
	z=0	$u_{g}=0,w_{g}=U_{gi}$
	z=L	$P_{g}=P_{atm}$
Cooling channel	$r=r_{t-c}$	$u_{c}=0,w_{c}=0$
	$r=r_{c}$	$\frac{\partial u_{c}}{\partial r}=0,\frac{\partial w_{c}}{\partial r}=0$
	z=0	$P_{c}=P_{atm}$
	z=L	$u_{c}=0,w_{c}={-U}_{ci}$

**Table 4:** Boundary conditions for the heat transport equations.

Domain	Position	Boundary Condition
Feed	r=0	$\frac{\partial T_{f}}{\partial r}=0$
	z=0	$T_{f}=T_{fi}$
	z=L	$\frac{\partial T_{f}}{\partial z}=0$
Membrane	z=0	$\frac{\partial T_{m}}{\partial z}=0$
	z=L	$\frac{\partial T_{m}}{\partial z}=0$
Stagnant WG	z=0	$\frac{\partial T_{g}}{\partial z}=0$
	z=L	$\frac{\partial T_{g}}{\partial z}=0$
Circulating WG	z=0 & z=L	Periodic
Cooling tube	z=0	$\frac{\partial T_{t}}{\partial z}=0$
	z=L	$\frac{\partial T_{t}}{\partial z}=0$
Cooling channel	$r=r_{c}$	$\frac{\partial T_{c}}{\partial r}=0$
	z=0	$\frac{\partial T_{c}}{\partial z}=0$
	z=L	$T_{c}=T_{ci}$

**Table 5:** Average temperature and concentration at feed–membrane interface of stagnant HF-WGMD module for different feed Reynolds numbers at $T_{fi}=50\;{}^{\circ}C$ and ${Re}_{c}=2300$.

	Feed Inlet Reynolds Numbers					
	460	1380	2300	2760	3680	4600
**Temperature [°C]**	45.1	47.2	47.9	49.5	49.6	49.7
$\mathrm{Concentration}\;[mol/m^{3}]$	3.53	3.9	4.04	4.38	4.42	4.44

**Table 6:** Average temperature and concentration at membrane–WG interface of circulating HF-WGMD module for different WG circulation Reynolds numbers at $T_{fi}=50\;{}^{\circ}C$, ${Re}_{f}=2760,$ and ${Re}_{c}=146$.

	WG Inlet Reynolds Numbers					
	441	1322	2204	2712	5424	8136
**Temperature [°C]**	31.9	32.4	34.1	23	22.9	22.9
**Concentration** $\left[mol/m^{3}\right]$	1.88	1.93	2.11	1.15	1.14	1.14

## Data Availability

The original contributions presented in this study are included in the article. Further inquiries can be directed to the corresponding author.

## Nomenclature

$a_{w}$	Water activity coefficient
$C_{p}$	Specific heat at constant pressure [J/(kg K)]
c	Concentration [mol/m^3^]
D	Diffusion coefficient [m^2^/s]
$D_{Kn}$	Knudsen diffusion coefficient [m^2^/s]
$D_{Or}$	Ordinary molecular diffusion coefficient [m^2^/s]
d	Diameter [m]
$d_{p}$	Pore diameter [m]
$h_{fg}$	Latent heat of vaporization [J/kg]
J	Product water flux [kg/m^2^h]
Kn	Knudsen number
k	Thermal conductivity [W/(m K)]
L	Module length [m]
M	Molecular mass [g/mol]
P	Pressure [Pa]
ppm	Concentration in parts per millions
q	Absorbed/expelled heat flux [W/m^2^]
R	Half of center-to-center distance [m]
r	Radius [mm]
Re	$\frac{\rho Ud_{f}}{\mu }$
$\bar{R}$	Universal gas constant [J/(mol K)]
STEC	Specific thermal energy consumption [kWh/m^3^]
T	Temperature [°C]
*U*	Flow stream mean velocity [m/s]
u	Velocity component in radial direction [m/s]
v	Specific volume [m^3^/kg]
W	Vapor content [kg_v_/kg_a_]
w	Velocity component in axial direction [m/s]
$x_{NaCl}$	Salt mole fraction
$x_{w}$	Water mole fraction
Subscripts	
a	Air
atm	Atmospheric
c	Coolant channel
f	Feed channel
g	Water gap
i	Inlet
m	Membrane
o	Outlet
r	Radial direction
sat	Saturation
t	Cooling tube
v	Vapor
w	Water
z	Axial direction
Greek symbols	
ε	Membrane porosity
μ	Fluid dynamic viscosity [Pa s]
ρ	Fluid density [kg/m^3^]
σ	Molecule collision diameter [m]
τ	Membrane tortuosity
